# Cross-sectional study and genotyping of rotavirus-A infections in ruminants in Kuwait

**DOI:** 10.1186/s12917-021-02944-4

**Published:** 2021-07-17

**Authors:** Nadra-Elwgoud M. I. Abdou, Qais A. H. Majeed, Ashraf A. Saad, Slavica Mijatovic-Rustempasic, Michael D. Bowen, Attia Samy

**Affiliations:** 1 Early Warning Center for Transboundary Animal Diseases-Gulf Cooperation Council, PAAFR, P.O. box 21422, 1307 Safat, Rabyia, Farwanyia, Kuwait; 2 Department of Medicine and Infectious Diseases, Faculty of Veterinary Medicine, Cairo University, 12211 Giza, Egypt; 3 Department of Science, College of Basic Education, PAAET, 23167, Aridyia, Farwanyia, Kuwait; 4 Virology lab., Veterinary Laboratories, PAAFR, 1307 Safat, Rabyia, Farwanyia, Kuwait; 5 Department of Virology, Animal Health Research Institute, 12618 Dokki, Giza, Egypt; 6 Viral Gastroenteritis Branch, Division of Viral Diseases, National Center for Immunization and Respiratory Diseases, Centers for Disease Control and Prevention, Atlanta, GA 30329 USA; 7 Department of Virology, Faculty of Veterinary Medicine, Cairo University, 12211 Giza, Egypt

**Keywords:** RVA, Immunochromatography, ELISA, RT-qPCR assay, G10P[11], Cattle, Sheep, Goats

## Abstract

**Background:**

Group A rotaviruses (RVA) are zoonotic pathogens responsible for acute enteritis in human and neonatal ruminants. This research aimed to determine the prevalence of RVA in ruminants (cattle, sheep, and goats) and investigate the circulating RVA genotypes in these animals in Kuwait. We conducted a cross-sectional study to detect RVA in ruminants, using an immunochromatography test (IC), direct sandwich ELISA test, and real-time RT-PCR (RT-qPCR) assay using fecal samples.

**Results:**

A total of 400 cattle, 334 sheep, and 222 goats were examined. The prevalence of RVA was 5.3, 1.2, and 2.3%, respectively, using IC. The ELISA test detected RVA from 4.3% of cattle, 0.9% of sheep, and 1.8% of goats. There was a significant association between the occurrence of diarrhea and the presence of RVA in bovine fecal samples (*p-*value = 0.0022), while no statistical association between diarrhea and the presence of RVA in fecal samples of sheep and goats was observed (*p-*value = 0.7250; *p-*value = 0.4499, respectively). Twenty-three of the IC-positive samples (17 from cattle, two from sheep, and four from goats) were tested using a RT-qPCR RVA detection assay targeting the NSP3 gene. The results showed that 21 of 23 IC-positive samples tested positive by RT-qPCR. Detection of RVA genotypes revealed that G10P[11] was the predominant strain in cattle (58.8%), followed by G8P[1] (11.7%). One sheep sample was genotyped as G8P[1]. In addition, G6P[1] and G6P[14] were detected in goat samples.

**Conclusion:**

The present study revealed that the IC was more sensitive in detecting RVA antigen in fecal samples than the ELISA test. A higher occurrence of RVA infection was observed in cattle than in sheep and goats. This study suggests that RVA might be a risk factor of diarrhea in bovine calves less than 2 weeks old. This research also demonstrates the circulation of RVA in sheep and goat populations in Kuwait. Finally, the G10P[11] RVA genotype was the most prevalent genotype identified from cattle samples.

**Supplementary Information:**

The online version contains supplementary material available at 10.1186/s12917-021-02944-4.

## Background

Rotavirus A (RVA) is the predominant viral gastroenteritis pathogen that infects both humans and animals [1]. It is endemic worldwide and results in an estimated 128,500 deaths and 258,173,300 diarrheal episodes among children under the age of 5 yearly [2]. RVA is the most critical species identified from ruminant diarrhea cases [3, 4]. It is usually reported in neonatal ruminant animals about 1–2 weeks of age. This is because milk feeding can provide a better survival environment for RVA across a broad spectrum of gastrointestinal pH levels and facilitate viral infection of the epithelial cells lining of the intestine. Thus, milk feeding could account for the higher susceptibility of non-weaned neonate animals to diarrhea [5].

Historically, RVA strains were typed using the genes of the outer capsid proteins VP7 (glycoprotein, G) and VP4 (protease-sensitive protein, P). Currently, RVA has 36 different G genotypes, G1-G36, and 51 different P genotypes, P[1]- P[51] [6]. The vast majority of bovine RVA genotypes detected during field studies have been G6, G10, and less commonly G8. These are usually associated with[5], P[11], and less often P[1] [7, 8]. The frequently reported genotype combinations in cattle are G6P[5], G6P[1] , and G10P[11] [9]. Genotypes G8P[1] and G8P[14] have been detected in lambs from Spain [10, 11], while G6P[11] has been reported as the predominant ovine strain in India [12]. Most of the ovine G-types (G10, G3, G6, and G9) circulate combined with P[1] , P[11] , P[14] , and P[9] [13]. Only limited data are available for caprine RVA genotypes, but G6P[1], G6P[14], G8P[1], G3P[3], and G10P[15] genotypes have been reported in goats globally [4].

In Kuwait, only one RVA survey investigated infection in dromedary camels. Al-Mutairi [14] detected a single RVA positive sample among 408 (0.2%) fecal samples using commercial-available EIA, while eight of 109 samples (7.3%) were RVA positive using RT-PCR assay. Of these eight positives, five samples were from four-month-old diarrheic animals and three samples were from three-year-old asymptomatic animals. The Kuwaiti camel rotavirus strain was identified as RVA/Camel-wt/KUW/s21/2010/G10P [15]. Subsequently, phylogenetic analysis showed similarities between the Kuwaiti camel strain and the ovine and bovine strains [15].

Continuous surveillance of the RVA disease in domestic animals is crucial for understanding the epidemiology and evolution of RVA strains in these animals. The aim of this study was to investigate the prevalence of RVA infections, its role in diarrhea, and to determine the dominant G and P genotypes in cattle, sheep, and goats in Kuwait.

## Results

The total number of animals on the visited farms was 18,422 (9365 cattle, 5428 sheep, and 3629 goats). Rectal fecal samples were randomly collected from 956 animals: 400 cattle, 334 sheep, and 222 goats.

### RVA detection by IC, ELISA and PCR

Using IC, RVA was detected in 21 (5.3%) of cattle, 4 (1.2%) of sheep, and 5 (2.3%) of goats. Using the ELISA test, RVA was identified in 17 (4.3%) of examined cattle, 3 (0.9%) of sheep, and 4 (1.8%) of goats (Table 1). Of these 30 samples, 18 were positive using both IC and ELISA (12 from cattle, two from sheep, and four from goats. Seventeen cattle samples were tested using RT-qPCR; all 17 returned positive results using both IC and RT-qPCR. Three samples were negative using the ELISA. This suggests that, in cattle samples, the IC was more sensitive in the detection of RVA than the ELISA test. For sheep and goat samples, six samples were positive for RVA using both IC and ELISA tests. These six samples were examined at the CDC using the RT-qPCR assay. Two of them were subsequently found negative (one sheep and one goat sample) (Table 1). A higher occurrence of RVA infection was observed in cattle than in sheep and goats (Table 1).

**Table 1:** RVA detected in the examined fecal samples from different ruminant animals by IC, ELISA, and qRT-PCR

Animal species	No of examined animals	IC+ (%)	ELISA+ (%)	IC+ ELISA+	# sent to CDC	IC + ELISA + qRT-PCR -	IC + ELISA - qRT-PCR +	IC+ ELISA+ qRT-PCR +	IC+ ELISA (not tested) qRT-PCR +
Cattle	400	21 (5.3)	17^a^ (4.3)	12/21	17^b^	0	3	12	2
Sheep	334	4 (1.2)	3 (0.9)	2/4	2	1	0	1	0
Goats	222	5 (2.3)	4 (1.8)	4/5	4	1	0	3	0

^a^Two cattle samples were not tested by ELISA test (17/398), ^b^Includes the 2 samples not tested by ELISA, (+) positive result, (−) negative result

### RVA association with diarrhea and different age groups

There was a significant association between the occurrence of diarrhea and the presence of RVA in fecal samples of cattle (*p-*value = 0.0022). No statistical association between diarrhea and identification of RVA in fecal samples of sheep and goats was observed (*p-*value = 0.7250; *p-*value = 0.4499, respectively; Table 2).

**Table 2:** Univariate analysis (χ^2^) for detection of RVA in ruminant animals using IC

Species	No of examined animals	Fecal Consistency				*p-*value
		Diarrheic (%)		Non- Diarrheic (%)		
		No. of Diarrheic animals	RVA positive (%)	No. of Non-Diarrheic animals	RVA positive (%)	
Cattle	400	127	13 (10.2%)	273	8 (2.9%)	0.0022
Sheep	334	111	1 (0.9%)	223	3 (1.3%)	0.7250
Goats	222	80	1 (1.3%)	142	4 (2.8%)	0.4499

Statistical analysis also identified age as a risk factor for RVA infection in cattle (*p-*value 0.00001). Calves ≤14 days old had the highest prevalence (9/47; 19.1%) followed by a prevalence of 9.8% (6/61) in calves aged 15–30 days old. In sheep and goats, age was not a statistically significant risk factor for RVA infection (*p-v*alue = 0.0832; *p-*value = 0.2545; respectively; Table 3).

**Table 3:** Distribution of positive RVA cases using IC in different age groups of the examined animals

	Cattle		Sheep		Goats	
	No. examined	Positive (%)	No. examined	Positive (%)	No. examined	Positive (%)
≤ 14 days	47	9 (19.1)	33	2 (6.1)	13	–
15–30 days	61	6 (9.8)	37	–	16	–
31–60 days	28	1 (3.8)	55	1 (1.8)	24	1 (4.2)
61–90 days	40	2 (5.0)	42	–	32	2 (6.3)
Over three months*	224	3 (1.3)	167	1 (0.6)	135	2 (1.5)
Total	400	21	334	4	222	5
*p-*value	0.0001		0.2545		0.0832	

### RVA genotypes

The G-types were identified in 95.2% (20/21) of RT-qPCR positive samples. G genotype could not be assigned to one of the bovine sample. Furthermore, P-types were identified in 90.5% (19/21) of RT-qPCR positive samples, with two cattle samples not typed (Table 4). In the cattle samples, 64% (11/17) of G genotypes were G10, 17.6% (3/17) were G6, and 11.7% (2/17) were G8. P-typing showed that 58.8% (10/17) were P[11], 17.6% (3/17) were P[1], and 11.8% (2/17) were P[5]. In cattle samples, the G and P combinations of the genotyped RVA showed that G10P[11] was the predominant strain (58.8%). This is followed by G8P[1] (11.7%). Then each of the G6P[1], G6P[5] and G6P[untyped] genotypes was reported in one sample (5.9%). One further G-P untyped sample was observed (Table 4).

**Table 4:** Results of RVA genotype combinations detected in cattle, sheep, and goats in Kuwait

Animal species	No of RVA Positives	Genotype combinations (%)							
		G10P[11]	G10P[5]	G6P[1]	G6P[5]	G6P[14]	G6P[nt*]	G8P[1]	Gnt*P[nt*]
Cattle (%)	17	10 (58.8%)	1 (5.9%)	1 (5.9%)	1 (5.9%)		1 (5.9%)	2 (11.7%)	1 (5.9%)
Sheep (%)	1							1 (100%)	
Goats (%)	3			2 (66.7%)		1 (33.3%)			
Total	21	10 (47.6%)	1 (4.8%)	3 (14.2%)	1 (4.8%)	1 (4.8%)	1 (4.8%)	3 (14.2%)	1 (4.8%)

* not typed

In small ruminants, G8 was the detected G-type in sheep and G6 in goats. P[1] was identified in both sheep and goats. Meanwhile, P[14] was detected in one goat sample (Table 4). The genetic combination identified from the sheep sample was G8P[1]. G6P[1] and G6P[14] were detected in 66.7, and 33.3% of goat samples, respectively (Table 4).

## Discussion

Field surveys of RVA infections with the characterization of disseminated genotypes in ruminants are useful in understanding RVA epidemiology and the geographical distribution of the different genotypes. Evidences from these surveys can be used to improve disease control programs [5, 9]. This study is the first to identify RVA genotypes in ruminants (cattle, sheep, and goats) in Kuwait. It is also the first to investigate the prevalence of RVA and its role as a risk factor of diarrhea in sheep and goats in Kuwait.

In our study, when confirmed using RT-qPCR results, the IC was more sensitive in the detection of RVA antigen in fecal samples than the ELISA test. The variation in sensitivity and specificity of different diagnostic methods to detect RVA in fecal samples from different ruminant animals can be exploited in different uses [5, 16]. Thus, for example, the sensitivity of rapid IC for the identification of RVA in feces may be acceptable as a pen-side test, especially when it is used on samples gathered from animals suffering from acute gastroenteritis as large quantities of RVA particles are typically discharged in feces [17].

Previous studies on RVA infections in Kuwait, using an ELISA test, found that RVA is among the leading causes of diarrhea in calves with a prevalence of 11.6 and 28.8%, respectively, from diarrheic neonatal and preweaned calves [18, 19]. In the present study, RVA was detected at a lower percentage, 5.3% using IC and 4.3% using the ELISA test, from both diarrheic and non-diarrheic cattle. If only diarrheic animals were considered, the percentage positive was higher (10.25% using IC test) compared to the overall prevalence of both diarrheic and non-diarrheic cattle (5.3% using IC test). Even then, the prevalence of RVA in the present study is still less than the 11.6–28.8% prevalence reported in previous surveys [18, 19]. Based on these results, it is likely that the prevalence could have been higher if the samples were collected only from young diarrheic calves. The variation in detection rates of RVA from calves’ specimens can depend on the different diagnostic methods used, hygienic measures, and management practices employed in the farms. Isolation of neonatal calves away from their dams and adult animals decreases the frequency of RVA infection in adults which can serve as non-diarrheic carriers [20, 21].

In the present study, RVA was detected in 1.2 and 0.9% of fecal specimens from sheep by IC and ELISA, respectively. For goat fecal samples, it was identified in 2.3 and 1.8% using IC and ELISA, respectively. The incidence of ovine and caprine RVAs have been reported from different countries around the world. Based on epidemiological conditions and diagnostic tests used, their prevalence rates varied [12, 16]. This study clarified that RVAs circulated in small ruminant populations reared in Kuwait.

There was a significant association (*p*-value = 0.0022) between diarrhea and the presence of RVA in fecal samples of cattle, with a higher prevalence in diarrheic (10.2%) compared to non-diarrheic cattle (2.9%). Previous investigations confirmed the importance of RVA as the predominant viral causative agent of diarrhea and identified RVA as a neonatal calf scours virus [5, 22]. Generally, it was believed that RVA diarrhea is mainly due to malabsorption [23]. However, there is also evidence that it is due to the action of, the RVA enterotoxin, NSP4 [23, 24]. Nine fecal samples (19.9%, 9/47) that were obtained from calves ≤14 days old were diarrheic and positive for RVA. This suggests that the majority of RVA diarrhea in cattle occurs in the first 2 weeks of age. This age group was reported by many studies as the most susceptible age to contract infection with RVA [5, 22, 23].

In the present study, there was no association between the occurrence of diarrhea in small ruminants and the detection of RVA in their fecal samples. Moreover, no statistically significant association between different age groups and the presence of RVA was found. This could be due to small numbers of identified RVA (9 samples) in this study from sheep and goats. Few studies have been done on small ruminants around the world in which RVA has been identified as a cause of diarrhea in newborn lambs and kids [11, 16, 25–27].

Bovine RVA G-typing in our positive specimens showed that G10 was the predominant genotype, followed by G6 and G8 in examined fecal samples of cattle. Previous studies have shown that G10 was prevalent amongst cattle populations in India and Ireland [28–30] while other reports showed that G6 was the most frequent isolate detected in cattle farms from the USA, Canada, Brazil, Iran, and New Zealand [8, 22, 31, 32]. Most researchers found that G8 was detected in low frequencies compared to other important bovine G genotypes (G10 and G6). However, investigators from Iran and New Zealand did not detect G8 in examined cattle populations [31, 32]. In contrast, another study from Tunisia confirmed that G8 as the most common G-type followed by G6, and the G10 genotype was absent [33]. Concerning P-typing results in examined cattle samples, P[11] was the most common P-type identified, followed by P[1] and P[5]. Previous studies from India [28] and Italy [34] identified P[11] as the most prevalent type in calves. However, another study reported that P[5] was the prominent type identified, followed by P[11] and P[1][8]. A study in Iran reported that G6 (55.3%), G10 (43.5%), P[5] (51.8%) and P[11] (27%) were the predominant genotypes detected [35].

In the present study, the most common G and P combination detected from cattle samples was G10P[11] (58.8%). Previous reports identified the prevalence rate of G10P[11] at 12.8% in USA [7], 15.3% in Iran [35], 81% in India [28], and 31.5% in Italy [34]. Other combinations reported in this study were G8P[1], G6P[1], G6P[5], and G6P[untyped]. Globally, G10P[11], G6P[5], and G6P[1] were reported as the most frequent RVA genotypes identified in calves [9].

The only ovine RVA genotype detected in this study was G8P[1]. G8P[1] has been reported as the leading cause of a diarrheal epidemic in two-months-old lambs in Spain [11] and recently was recovered from sheep in Turkey [36]. The genetic combinations G6P[1] and G6P[14] were identified from the caprine samples. The G6P[1]genotype combination was the most common genotype reported in goats all over the world (for example, in Turkey [36], Italy [37], and Bangladesh [38]). However, G6P[14] was previously detected from goats in South Africa [39].

Several studies sporadically detected new human RVA genotypes, such as G6, G8, and G10 associated with P-types ([1], [3], [9], [10], [11], and [14]) [40, 41]. Many of these new human RVA genotypes are frequently reported in animals, suggesting that these genotypes are of animal origin. These RVA genotypes might have been introduced to human populations through interspecies transmission and/or genetic reassortment of rotavirus strains [41, 42]. To the best of our knowledge, only one previous study describes the human RVA genotypes in Kuwait [43]. The human RVA genotypes reported in Kuwait (G1, G9, G2, G4, and G3 associated with P[8]) were not detected in the examined ruminant animals in our study. On the other hand, G6P[1], G6P[14], and G8P[1], which were detected in this study, have been globally identified in cases of diarrheic children with a history of visiting animal quarters [44, 45]. Furthermore, G6P[14] was detected from the stool of a child in Egypt and showed great similarity to the ovine and simian origin [46]. Additionally, Ghosh et al. [47] conducted full genomic sequencing of two human G2P[4] strains in Bangladesh and found that the VP3 gene of strain MMC88 was most closely related to a local caprine strain. As a result, comparisons of the genotypes and complete genomes of human strains with those of co-circulating animal RVA strains could provide a better understanding of the natural reassortment events that occur between human and animal RVAs [47]. Finally, direct animal-to-human transmission of RVA in Kuwait and, more widely the Gulf area, requires further studying and surveillance.

## Conclusions

The present study suggests that RVA might be a risk factor of diarrhea in bovine calves less than 2 weeks old; this needs further investigations that consider other risk factors. G10P[11] was the most predominant genotype diagnosed in cattle. This research demonstrates the circulation of RVA in sheep and goat populations in Kuwait but suggests that it is not a diarrheal risk factor in these animals. RVA strains reported in this study in ruminant animals are yet to be detected in humans in Kuwait. However, they have been detected in humans in some of the Middle East countries.

## Methods

### Study design and sample collection

A cross-sectional study was conducted during the period extending from October 2014 to September 2015. Farms involved in the study were visited once, and fecal samples were collected from different age groups, independent of the appearance of clinical signs (diarrhea). Farms were either single- or mixed-species. Epi Info*™* sample size calculator was used to determine the sample size for each visited farm. Sampling was based on the number of animals in the examined farm. Systematic random sampling was used to select animals for this study.

The rectal fecal samples (about 5–10 g of feces) were taken directly from the rectum or immediately after defecation. The collected fecal samples were placed in clean containers and tagged with the data of the sampled animal, such as species, sex, age, and health status, in addition to the date of sampling, owner’s name, and location of the farm. The containers containing samples were placed in an icebox and taken to the laboratory as soon as possible. In the laboratory, fecal samples were classified according to their consistency- diarrheic (pasty/watery), or non-diarrheic (normal). Then the samples were divided into two portions, one for a rapid screening test using an immunochromatography test, and the other portion was kept at − 20^0^ C for further examination by ELISA and PCR.

This study adheres to the ARRIVE Guidelines for reporting animal research. A completed ARRIVE guidelines checklist is included in Supplementary file 1.

### Immunochromatography test

A rapid IC detection kit (BoviD-4, BioNote, Gyeonggi-do, Republic of Korea) was used as a screening test for the detection of the presence of antigens for four pathogens (*Cryptosporidium*, Bovine RVA, Bovine coronavirus, and *E. coli*). All the 956 samples collected were tested using the rapid test, as described by the manufacturer.

### ELISA test

All fecal samples were examined with a direct, sandwich, double-well, ELISA kit for the detection of RVA antigen (Bio-X Diagnostics, Rochefort, Belgium). The optical densities were read at 450 nm using a microplate reader (BioTek Instrument, Inc. Winooski, USA). The validation, calculation, and interpretation of the results were performed according to the manufacturer’s instructions.

### RNA extraction

The *AccuPrep®*Viral RNA Extraction Kit (Bioneer Corporation, Daedeokgu, Daejeon, Republic of Korea) was used as described by the manufacturer for the extraction of RVA RNA. Twenty- three RNA extracts (17, 2, 4 isolated from cattle, sheep, and goats, respectively) were sent to the Centers for Disease Control and Prevention (CDC), Atlanta, USA, for PCR and genotyping.

### RT-PCR and genotyping

The samples were tested by a real-time RT-PCR (RT-qPCR) RVA detection assay targeting the NSP3 gene [48]. RT-PCR amplification and Sanger sequencing of the VP7 and VP4 genes were performed as described previously [49] with the following modifications: 1) amplicons generated for sequencing were analysed and purified on E-gel® SizeSelect cartridges (ThermoFisher Scientific) following the manufacturer’s instructions; and, 2) purification of cycle-sequenced products was performed with a commercially available BigDye XTerminator*™* Purification Kit (ThermoFisher Scientific), following the manufacturer’s protocol.

Genotypes were assigned based on comparison of 835 bp VP7 and 858 bp VP4 sequences generated using 9con1L (bindsVP7 gene bp 37–56)/VP7R (914–933) [49, 50] primers and con3 (binds VP4 gene bp 11–32)/con2 (868–887 bp) primers [49], respectively. RVA genotypes were assigned using BLAST searches of the GenBank Nucleotide Collection (nr/nt) database (http://bla st.ncbi.nlm.nih.gov/Blast.cgi) to establish sequence identity to reference genotypes [50]. Full genome sequences were obtained following a protocol previously described [50].

### Statistical analysis

A chi-square test (*χ*^2^) was used to identify the role of RVA as a risk factor of occurrence of diarrhea in cattle, sheep, and goats in Kuwait. The statistical relationship between different age groups and the prevalence of RVA infection in these animals was found in a univariate setting. The *χ*^2^ test and *p*-value at the 95% confidence interval were calculated using the Statistix 10© statistical analysis software.

## Supplementary Information

**Additional file 1.** The ARRIVE Guidelines Checklist.

**Additional file 2.** Sample Collection Approval Form.

## Data Availability

The datasets used and/or analysed during the current study are available from the corresponding author on reasonable request.

## Abbreviations

IC: Immunochromatography test
ELISA: Enzyme Linked Immunosorbent Assay
RT-qPCR: Real-time quantitative Reverse Transcription Polymerase Chain Reaction
G: Glycoprotein
P: Protease-sensitive protein
*χ*^2^: Chi-square test
