# Supplementary material for: Cross-sectional study and genotyping of rotavirus-A infections in ruminants in Kuwait
Source: BMC Vet Res. 2021 Jul 17;17:245. doi: 10.1186/s12917-021-02944-4 (PMC8286158; doi:10.1186/s12917-021-02944-4)
Supplement: Supplementary file 2 — Additional file 2. Sample Collection Approval Form. [file 12917_2021_2944_MOESM2_ESM.docx]

Supplementary-1

Copy of the Sample Collection Approval form (in Arabic) and English translated copy

**Cross-sectional study and genotyping of Rotavirus-A infections in ruminants in Kuwait**

**-Research Article-**

## Nadra-Elwgoud M.I. Abdou^1,2*^, Qais Majeed^3^, Ashraf Saad^4,5^, Slavica Mijatovic-Rustempasic^6^, Michael Bowen^6^ and Attia Samy^4,7^

^1^GCC-Early Warning Center, PAAFR, Rabyia, Postal code 1307, Kuwait. ^2^Department Medicine and Infectious Diseases, Faculty of Veterinary Medicine, Cairo University, Giza, Post code 12211, Egypt. ^3^Department Science, PAAET, College of Basic Education, Aridyia, Post code 23167, Kuwait. ^4^Virology lab., Veterinary Laboratories, PAAFR, Rabyia, Postal code 1307, Kuwait. ^5^Department Virology, Animal Health Research Institute, Dokki, Post code 12618, Egypt.^6^Viral Gastroenteritis Branch, Division of Viral Diseases, National Center for Immunization and Respiratory Diseases, Centers for Disease Control and Prevention, Atlanta, Georgia 30333, USA. ^7^Department Virology, Faculty of Veterinary Medicine, Cairo University, Giza, Post code 12211, Egypt.

نموذج الموافقة على جمع العينات

أوافق أنا السيد...................................بصفتي مالك/ مدير مزرعة.......................................على التصريح للفريق البحثي الخاص بالمشروع رقم (KFAS-Award Number 2012-1207-04) وعنوانه**:** دراسـة وبائيـة عن مرض الكربيتوسبوريديم والعـدوى بفيروس الروتا في الثروة الحيوانية بدولة الكويت، بتجميع عينات من الحيوانات (إبقار، أغنام، ماعز، جمال) المربى بالمزرعة.

وقد أوضح الباحثين انه سيتم تجميع عينات براز فقط (حوالي5-10 جرام) مباشرة من فتحة الشرج او بعد التبرز مباشرة، وذلك مع التأكيد على إتباع جميع الإرشادات الدولية والوطنية لرعاية واستخدام الحيوانات. وأكد الباحثين كذلك الحفاظ على خصوصية بيانات المزرعة وأن البيانات ستتم مشاركتها فقط للأغراض البحثية.

التوقيع:

التاريخ:

Translated Copy of

Sample collection approval form

I, hereby Mr. ................................... as owner / manager of farm name ..................................... give permission for the researcher of the project No. (KFAS-Award No. 2012-1207-04) entitled “Epidemiological Studies on Cryptosporidium and Rotavirus Infection in Livestock in the State of Kuwait”, by collecting samples from animals (cows, sheep, goats, camels) raised on the farm.

The researchers declares that rectal fecal samples (about 5-10 grams) will be collected directly from the rectum or immediately after defecation and international and national guidelines for the care and use of animals will be followed. The researcher also guaranteed that the data on the farm will remain unidentifiable and that the data will only be shared for research purposes.

Signature:

Date:
